# Child sexual abuse in religiously affiliated and secular institutions: a retrospective descriptive analysis of data provided by victims in a government-sponsored reappraisal program in Germany

**DOI:** 10.1186/1471-2458-14-282

**Published:** 2014-03-27

**Authors:** Nina Spröber, Thekla Schneider, Miriam Rassenhofer, Alexander Seitz, Hubert Liebhardt, Lilith König, Jörg M Fegert

**Affiliations:** 1 Department of Child and Adolescent Psychiatry/Psychotherapy, University Hospital Ulm, Steinhoevelstrasse 5, 89075 Ulm, Germany; 2 Soon Systems GmbH, Syrlinstrasse 5, 89073 Ulm, Germany; 3 Department of Special Education, University of Education Ludwigsburg, Reuteallee 46, 71634 Ludwigsburg, Germany

**Keywords:** Child sexual abuse, Religiously affiliated residential care centres, Non-religiously-affiliated residential care centres, Psychosocial consequences, Prevention

## Abstract

**Background:**

The disclosure of widespread sexual abuse committed by professional educators and clergymen in institutions in Germany ignited a national political debate, in which special attention was paid to church-run institutions. We wanted to find out whether the nature of the abuse and its effect on victims differed depending on whether the abuse had been experienced in religiously affiliated versus secular institutions.

**Methods:**

In 2010, the German government established a hotline that victims could contact anonymously to describe their experiences of sexual abuse. The information provided by callers was documented and categorized. Our analysis looked at a subset of the data collected, in order to compare the nature of the abuse experienced at three types of institutions: Roman Catholic, Protestant, and non–religiously affiliated. Non-parametric tests were used to compare frequency distributions, and qualitative data were analyzed descriptively.

**Results:**

Of the 1050 victims in our sample, 404 had been in Roman Catholic, 130 in Protestant, and 516 in non-religious institutions. The overall mean age at the time of reporting was 52.2 years. Males (59.8%) outnumbered females. Victims who had been in religiously affiliated institutions were significantly older than those who had been in secular institutions. Almost half the victims had been abused physically as well as sexually, and most victims reported that the abuse had occurred repeatedly and that the assaults had been committed by males. Patterns of abuse (time, type, and extent), and the gender of the offenders did not differ between the three groups. Intercourse was more frequently reported by older victims and by females. Similar percentages of victims in all groups reported current psychiatric diagnoses (depression, anxiety disorders, PTSD). Significantly more victims from Protestant institutions reported having current psychosocial problems.

**Conclusion:**

The results suggest that child sexual abuse in institutions is attributable to the nature of institutional structures and to societal assumptions about the rights of children more than to the attitudes towards sexuality of a specific religion. The exploratory data arising from this study may serve as a starting point for building hypotheses, and may point the way toward improvements in prevention and intervention strategies.

## Background

Child sexual abuse is a widespread problem in many societies. In Germany, studies have estimated that 6% to 13% of children overall are sexually abused [1,2], which is consistent with the rates reported in global studies [3-5]. Much of the research, however, has focused on the situation within families. Understanding of the characteristics of sexual abuse in institutions is lacking, and the prevalence of the problem in these settings is unknown [6].

In 2010, the disclosure of several cases of sexual abuse in institutions in Germany, particularly in schools operated by the Roman Catholic Church, ignited a national political debate and led to public outrage over the extent of abuse committed by professional educators. The German scandal was only one of a series of similar scandals in the United States, the United Kingdom, Belgium, the Netherlands and Ireland, all of which led to public debates and to the establishment of committees for compiling reports of sexual abuse in institutions [7-12]. Most of these reports addressed problems that had occurred decades earlier (between 1940 and 1980), and revealed that children, particularly boys, who were raised in institutions were at high risk of maltreatment [8,13]. Offenders were predominantly male, and were mostly educators, priests or other members of religious communities, and institutional staff [8]. Some offenders had abused multiple children over many years. The type of sexual abuse ranged from voyeurism to rape, and was sometimes accompanied by physical or emotional abuse or neglect.

A common scheme seems to be that sexual abuse in institutions is characterized by exploitation of the hierarchic structures of power and dependence that typically define the relationship between youth and their caregivers [14]. While all institutional environments support relationships that are marked by a certain degree of dependence, some are more susceptible to aberrations. The risk has been found to be higher in closed systems, as power and proximity generate a behavioural pattern that may facilitate sexual abuse. The term “total institution” [15,16] was defined to denote the fact that a child’s life in an institution is strictly controlled by a single authority, and residents are secluded from the outside world [16]. Obviously, it is easier to sever ties to some types of institutions (e.g., sports clubs) than others (e.g., schools or residential care centres) [17]. However, open systems too can facilitate sexual abuse, as they allow easy access to children who are not under constant supervision [18].

Victims of sexual abuse are very often threatened with negative consequences in order to keep them silent [19]. Years before the recent international focus on the problem, Summit [20] identified factors contributing to silence, which include secrecy, helplessness, entrapment, accommodation, and the fear of not being taken seriously when revealing abuse. Another factor is that children and adolescents are often strongly desirous of belonging to a social group and will idealize it; if a child then witnesses a member of that group committing an offense, the child’s trust in that ideal is broken. If other members of the group are aware of what was witnessed, the child may choose to deny, conceal, or justify the incident out of a fear of being excluded. Peer pressure thus may facilitate child abuse and complicate attempts to uncover the truth [21]. It has been found that even when authorities followed up on reports of maltreatment or sexual abuse, the act was commonly justified as a required disciplinary measure or as being excusable due to the offender being under high stress [22]. In most cases of sexual abuse connected to institutions or organizations, there were no severe consequences for the offenders [23,24]. Sometimes they were merely transferred, in some cases to another country, giving them the opportunity to continue to prey on children in a new environment [7,12,13,25]. This was particularly true for Roman Catholic institutions, due to the consolidated global organization of the Roman Catholic Church.

Although prevalence estimates of child sexual abuse in religiously – affiliated and religiously – non – affiliated institutions are unknown, the inquiry into sexual abuse in Germany paid special attention to institutions that were religiously affiliated, for several reasons. First, most residential care in Germany was traditionally provided by either the Roman Catholic or the Protestant church, and public awareness of the problem of child sexual abuse began following the revelations of students at a school run by the Jesuits. Second, while institutions within the Protestant church have been implicated in abuse, the number and severity of accusations are significantly higher for the Roman Catholic Church [26], which is seen as carrying particular risk factors due to the requirement of celibacy within the priesthood which may offer an opportunity for paedophiles to both conceal and satisfy their sexual preferences [27]. However, non–religiously affiliated institutions such as private boarding schools and state-run care facilities have received public attention as well, and authorities have begun to address and prosecute cases of maltreatment and sexual abuse in those environments [28]. One prominent case has been the private boarding school of Odenwaldschule, a closed system in which the sexual exploitation of students continued for three decades in the name of educational reform [29,30]. The educators there, while preaching self-determination, were not only abusing their wards but had set up a perfidious system of concealment and oppression that enabled ongoing abuse.

While resilience to and recovery from traumatic events are certainly possible [31], there is evidence that for many victims, child abuse can result in severe psychiatric disorders such as depression, anxiety, and post-traumatic stress disorder [32] and can have a significant and long-term impact on the ability to adjust to adult life [33]. In a study of 274 victims of sexual abuse in Ireland, the life prevalence rate for a psychiatric disorder was found to be 80% [34]. An important question is whether the consequences of sexual abuse differ if the abuse was experienced in a religiously affiliated rather than a secular institution, as the former could additionally have an impact on the victim’s spirituality and religiosity [35]. When the abuse is committed by someone whom the victim not only trusts but associates with religious values, the sense of betrayal and powerlessness may be exacerbated [36]. The intensity and destructive effects of the trauma associated with the abuse by a clerical superior may be directly related to the emotional bond the victim feels with the offender; a bond that is grounded in factors that are described as “spiritual” but which here have become toxic [37,38].

### Establishment of a reporting system in Germany for victims of abuse

When the scandal of sexual abuse in Germany broke, a number of vocal victims’ groups launched claims against several institutions as well as the state. However, it was recognized that the views of those groups might not be completely representative, since many victims are unable or unwilling to become politically involved. Accordingly, the government established two political entities to examine the problem. One was a Round Table committee, titled “Child Sexual Abuse in Relationships of Dependence, and Imbalance of Power in Private and Public Institutions and Families”, which was chaired by the federal ministries responsible for family matters, justice, and education and whose task was to develop recommendations and strategies concerning support for victims, prevention of future abuse, education of professionals, and judicial questions. The other was an appointee with the title of “Independent Commissioner for the Reappraisal of Child Sexual Abuse”, whose tasks were to gather information about past cases of child sexual abuse in both institutions and families and to develop a set of recommendations for Parliament and the Round Table regarding the provision of services for victims. These two entities set up an anonymous reporting system that could be accessed through various contact points: a hotline telephone number, a mailing address, and an email address. In addition, a web site was set up to keep victims informed about the results and outcomes.

The aim of the present study was to compare the impact on victims of sexual abuse in church-run versus secular institutions with regard to a number of factors. To achieve this, we used the data that had been gathered through the reporting system established by the Round Table and the Independent Commissioner. We compared three types of institutions: Roman Catholic, Protestant, and non–religiously affiliated, which collectively represent the majority of residential care institutions in Germany. In light of the review of the literature above, we wanted to find out whether the nature of the abuse and its effect on victims differed between these three groups, and whether those who had experienced sexual abuse in religiously-affiliated institutions would show more difficulties adjusting to adult life than those who had been abused in secular institutions. To our knowledge, this study is the first to look at these comparisons.

## Methods

### Collection of the data

Our research group was responsible for collecting and analyzing the testimonials provided by victims and for providing the findings to the Independent Commissioner, who incorporated them into her recommendations to Parliament and the Round Table. The development of this system involved a balancing of methodological requirements on the one hand, and practicability and ethical responsibility toward victims on the other. The hotline was staffed by more than 60 therapists and counsellors who were experienced in the domains of child abuse, neglect, and sexual violence. Callers to the hotline were allowed to control the conversation and to choose what to talk about. Although standardized interviews would have been preferable with respect to statistical methodology, it was felt to be more important that victims be able to speak about whatever they themselves felt to be relevant. Another consideration was the fact that even 20 years after the fall of the Berlin Wall, many Germans are highly suspicious of revealing their private affairs to state establishments. The guarantee of anonymity and the assurance of no initiation of any criminal investigation were regarded as necessary measures to instil trust.

All those who contacted the hotline were guaranteed anonymity. If the callers agreed, the hotline staff documented their experiences and messages, using a web-based template that provided standardized selections for a range of aspects including demographic data, context of the abuse, when the abuse had occurred, its frequency, whether it was still ongoing, and the prevalence and nature of any current mental health disorders or problems in psychosocial functioning. These categories were chosen based on knowledge of experts in the domain of child sexual abuse, on the literature, and on what information would be most useful in the development of new policies. In addition, free-text fields were provided for the entry of aspects that were not included in the pre-determined categories (e.g., offenders’ strategies). The establishment of the reporting system, data collection, and the political process in Germany are described more detailed elsewhere [39,40].

### Statistical methods

Descriptive statistics are presented as mean (± standard deviation) and frequency (percentage). Frequency distributions of the three institutional groups with respect to socio-demographic data, nature of the abuse, number of incidents, number of offenders, gender of offenders, current mental health disorders, and current problems in psychosocial functioning were compared using the Kruskall-Wallis test. Pairwise comparisons of significant differences of frequency distribution were calculated using the Mann–Whitney U-test. Data in the free-text fields are presented descriptively. All analyses were conducted using SPSS, version 18.0. Findings of p-values of < 0.05 were considered statistically significant. As the analyses were exploratory, no adjustment for multiple comparisons was made.

### Ethical considerations

The Ethics Review Board of the University of Ulm approved this research project (reference number 99/10, 4.5.2010).

## Results

### Population studied

Between May 2010 and August 2011, the Independent Commissioner received 7,565 calls and 3,062 letters or e-mails, from which 6,300 analysable data sets were obtained. These were categorized according to the caller’s or writer’s connection with the issue of sexual abuse: victim, associate of a victim, offender, associate of an offender, or other. See Table 1.

**Table 1:** Distribution of distinct groups of people who contacted the hotline of the independent commissioner

	**Letters/emails**	**Calls**	**Total**
**Total**	**N = 1575**	**N = 4725**	**N = 6300**
Victims	1074 (68.2%)	3134 (66.1%)	4208 (66.8%)
Associate of a victim	244 (15.5%)	738 (15.6%)	982 (15.6%)
Offenders	6 (0.4%)	34 (0.7%)	40 (0.6%)
Associate of an offender	3 (0.2%)	22 (0.5%)	25 (0.4%)
Other	248 (15.7%)	797 (16.9%)	1.045 (16.6%)

Our analyses looked at the victim data only (N = 4208). Of that group, 1050 (25%) individuals indicated that the abuse had taken place within an institutional context. We further categorized this subset according to what type of institution was involved: Roman Catholic (N = 404), Protestant (N = 130), or non-religious (N = 516), with the first two categories comprising both schools and residential care centres and the third comprising places such as state residential child care facilities. See Table 2.

**Table 2:** Overview of institutions

**Institutions**	**Roman Catholic context**	**Protestant context**	**Non- religiously affiliated context**
**Total**	**N = 404**	**N = 130**	**N = 516**
Schools	86 (21.3%)	14 (10.8%)	165 (32.0%)
Residential care centers	94 (23.3%)	25 (19.2%)	232 (45.0%)
Not specified	224 (55.4%)	91 (70.0%)	119 (23.1%)

**Figure 1:**
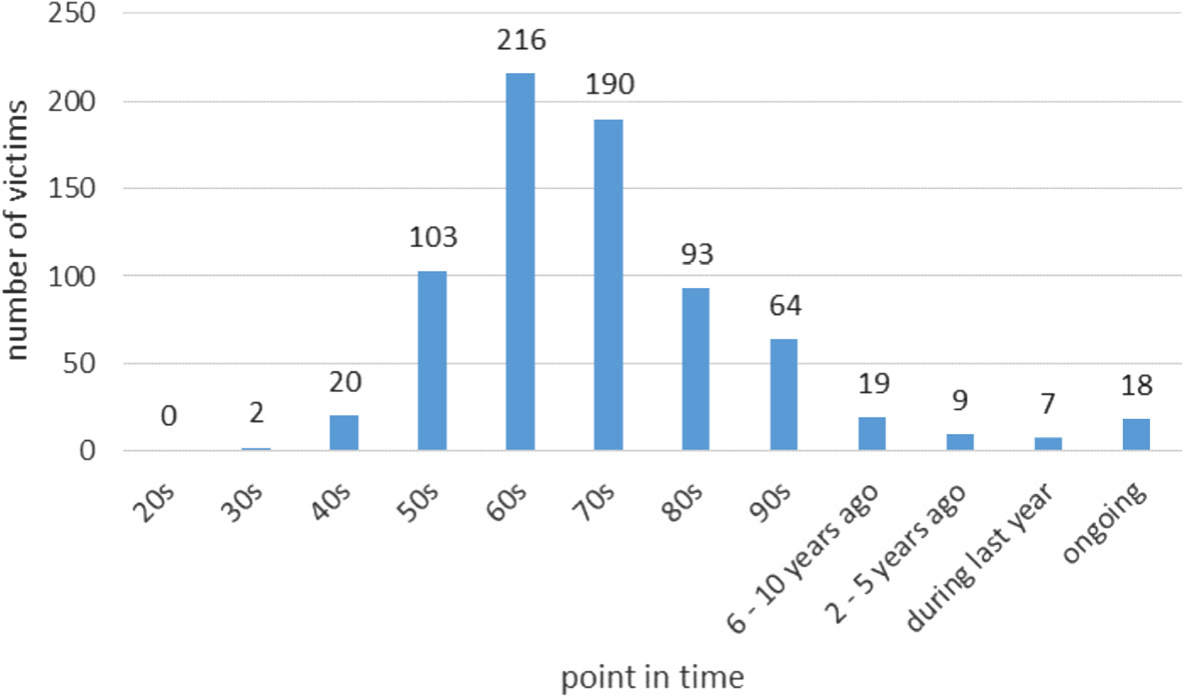
Number of victims (N = 723) and reported points in time when sexual abuse has occurred.

At the time of contact, the average age of victims was 52.2 years (SD = 13.5; range: 12–89 years). Male respondents (N = 614; 59.8%) outnumbered females (N = 412; 40.2%). The majority (N = 781; 90.7%) were living in the western federal states of Germany, and 68.7% N = 409) were living in urban environments. Only a minority (N = 191; 18.2%) had never been married; the remainder were either married, divorced/separated, widowed, or living common law. Of those who identified when the abuse had taken place, the majority reported that it had occurred between 1950 and 1980 (see Figure 1).

Table 3 presents demographic characteristics for each group. There was no significant age difference between victims in the Roman Catholic and the Protestant groups (p = 0.536), but victims in each of these groups were older than those in the secular group (p < 0.001 for both). There were significantly more males in the Roman Catholic group than in either the Protestant or the secular group (p < 0.001 for both), and significantly more females in the Protestant group than in either the Roman Catholic (p < 0.001) or the secular group (p = 0.043). No significant group differences were seen for marital status, education, and urban or rural environment; however, the lack of data for these three measures makes interpretation difficult.

**Table 3:** Demographic characteristics of victims dependent on context groups

	**Roman Catholic context**	**Protestant context**	**Non-religiously affiliated context**	**p-value**^**b**^
	**(N = 404**^**a**^**)**	**(N = 130**^**a**^**)**	**(N = 516**^**a**^**)**	
**Age**	**N = 327**	**N = 105**	**N = 392**	**.000**
	Missing data:	Missing data:	Missing data:	
	77 (19.1%)	25 (19.2%)	124 (24.0%)	
Range	19 – 89	34 – 78	12 – 79	
Mean (SD)	54.9	56.5	49.1	
	(SD = 13.0)	(SD = 8.8)	(SD = 14.2)	
**Gender**	**N = 394**	**N = 128**	**N = 503**	**.000**
	Missing data:	Missing data:	Missing data:	
	10 (2.5%)	2 (1.5%)	13 (2.5%)	
Male	275 (69.8%)	58 (45.3%)	280 (55.7%)	
Female	119 (30.2%)	70 (54.7%)	223 (44.3%)	
**Location**	**N = 332**	**N = 107**	**N = 422**	**.000**
	Missing data:	Missing data:	Missing data:	
	72 (17.8%)	23 (17.7%)	94 (18.2%)	
Western federal states	322 (79.7%)	101 (77.7%)	358 (60.4%)	
Eastern federal states	10 (2.5%)	6 (4.6%)	64 (12.4%)	
**Urban or rural environment**	**N = 230**	**N = 76**	**N = 289**	**.788**
	Missing data:	Missing data:	Missing data:	
	174 (43.1%)	54 (41.5%)	227 (44.0%)	
Urban	160 (39.6%)	54 (41.5%)	195 (37.8%)	
Rural	70 (17.3%)	22 (16.9%)	94 (18.2%)	
**Marital status**	**N = 212**	**N = 73**	**N = 234**	**.408**
	Missing data:	Missing data:	Missing data:	
	192 (47.5%)	57 (43.8%)	282 (54.7%)	
Single	74 (34.9%)	28 (38.4%)	89 (38.0%)	
Married	83 (39.1%)	22 (30.1%)	77 (32.9%)	
Divorced/separated	37 (17.4%)	21 (28.8%)	37 (15.8%)	
Widowed	7 (3.3%)	1 (1.4%)	9 (3.8%)	
Living with a domestic partner	11 (5.2%)	1 (1.4%)	22 (9.4%)	
**Education**	**N = 155**	**N = 46**	**N = 180**	**.924**
	Missing data:	Missing data:	Missing data:	
	249 (61.6%)	84 (64.6%)	336 (65.1%)	
A-levels	70 (45.2%)	21 (45.7%)	76 (42.2%)	
General certificate of secondary education	14 (9.0%)	5 (10.9%)	7 (3.9%)	
Certificate of secondary education	33 (21.3%)	11 (23.9%)	44 (24.4%)	
Certificate of school for special education	26 (16.8%)	4 (8.7%)	31 (17.2%)	
No graduation	2 (1.3%)	2 (4.3%)	4 (2.2%)	

ªTotal of whole context group; see detailed number of victims that referred to single topics in the table.

^b^Kruskall-Wallis-Test with statistically significant level p < .05.

### Characteristics of the abuse

There were no significant group differences with respect to the nature of the abuse (p = 0.149), when it had occurred (p = 0.515), the number of incidents (p = 0.421), or the gender of the offenders (p = 0.369). Overall, between 30% and 50% of victims described touching incidents (genitals or other parts of the body), while actual penetration/intercourse was reported by 49.0%, 57.4%, and 55.1% in the Roman Catholic, Protestant, and secular groups, respectively. For almost all victims (≥95.0%), the abuse was in the past, with just 2.1% in the Roman Catholic group and 2.9% in the secular group describing it as ongoing. Most victims (around 90%) have been abused more than once, in some cases repeatedly and over a long period of time. The majority of offenders had been male: 85.9%, 86.7%, and 82.6% for the Roman Catholic, Protestant, and secular groups, respectively. 611 (58.2%) victims had experienced child sexual abuse in different settings. See Table 4.

**Table 4:** Characteristics of child sexual abuse dependent on context groups

	**Roman Catholic context**	**Protestant context**	**Non-religiously affiliated context**	**p-value**^**b**^
	**(N = 404**^**a**^**)**	**(N = 130**^**a**^**)**	**(N = 516**^**a**^**)**	
**Type of sexual abuse**	**N = 200**	**N = 61**	**N = 245**	**.149**
	Missing data:	Missing data:	Missing data:	
	204 (50.5%)	69 (53.1%)	271 (52.5%)	
Non- physical acts	7 (3.5%)	2 (3.3%)	5 (2.0%)	
Physical acts (touching body or genitals)	97 (48.5%)	19 (31.1%)	105 (42.9%)	
Penetration/intercourse	98 (49.0%)	35 (57.4%)	135 (55.1%)	
**Time of abuse**	**N = 374**	**N = 120**	**N = 466**	**.515**
	Missing data:	Missing data:	Missing data:	
	30 (7.4%)	10 (7.7%)	50 (9.7%)	
Ongoing	11 (2.9%)	0 (0.0%)	10 (2.1%)	
Ongoing and past	8 (2.1%)	2 (1.7%)	9 (1.9%)	
Past	355 (94.9%)	118 (98.3%)	447 (95.1%)	
**Quantity of sexual abuse**	**N = 311**	**N = 104**	**N = 373**	**.421**
	Missing data:	Missing data:	Missing data:	
	93 (23.0%)	26 (20.0%)	143 (27.7%)	
Once	28 (9.0%)	9 (8.7%)	31 (8.3%)	
Multiple	94 (30.2%)	24 (23.1%)	111 (29.8%)	
Repeatedly over a long time	189 (60.8%)	71 (68.3%)	231 (61.9%)	
**Gender of offender**	**N = 341**	**N = 113**	**N = 408**	**.369**
	Missing data:	Missing data:	Missing data:	
	63 (15.6%)	17 (13.1%)	108 (20.9%)	
Male	293 (85.9%)	98 (86.7%)	337 (82.6%)	
Female	29 (8.5%)	2 (1.8%)	31 (7.6%)	
Both	19 (5.6%)	13 (11.5%)	40 (9.8%)	

ªTotal of whole context group; see detailed number of victims that referred to single topics in the table.

^b^Kruskall-Wallis-Test with statistically significant level p < .05.

Since significant group differences were seen with regard to victim gender and age, we examined the influence of these two variables. For age, we split the sample into three age groups: < 40 years, 40–59 years, and ≥ 60 years. Females and individuals in the youngest age group were more likely to report that penetration/intercourse had taken place (p = 0.038 and <0.001, respectively) and that they were still experiencing current abuse (p < 0.001 for both). These findings indicate that for some victims, the abuse suffered during childhood may be associated with involvement in abusive relationships in adulthood.

Table 5 shows the gender of offenders in relation to the gender of victims. Due to the small numbers, the results are merely descriptive. As mentioned earlier, there were far more male offenders than female in all groups. Cases involving a female offender and a male victim were seen most often in the Roman Catholic group (N = 88; 38.1%), far less often in the secular group (N = 24; 11.7%), and not at all in the Protestant group.

**Table 5:** Specification of gender of offenders and gender of victims dependent on context groups

	**Roman Catholic context**		**Protestant context**		**Non-religiously affiliated context**	
	**(N = 404**^**a**^**)**		**(N = 130**^**a**^**)**		**(N = 516**^**a**^**)**	
	**Gender of victims**		**Gender of victims**		**Gender of victims**	
**Gender of offender(s)**	Male	Female	Male	Female	Male	Female
Male	198 (85.7%)	21 (20.4%)	45 (83.3%)	52 (88.1%)	161 (78.5%)	169 (86.7%)
Female	88 (38.1%)	8 (7.8%)	0 (0.0%)	2 (3.4%)	24 (11.7%)	7 (3.6%)
Both	12 (5.2%)	7 (6.8%)	9 (16.7%)	5 (8.5%)	20 (9.8%)	19 (9.7%)
**Total of victims referring to this topic**	231 (100.0%)	103 (100.0%)	54 (100.0%)	59 (100.0%)	205 (100.0%)	195 (100.0%)

ªTotal of whole context group; see detailed number of victims that referred to single topics in the table.

Table 6 shows the rates of other types of abuse that were inflicted. The rates were similar across groups for physical abuse (44.4% to 47.5%), psychological abuse (38.1% to 39.4%), and emotional neglect (14.1% to 16.2%).

**Table 6:** Additional physical, psychological and emotional neglect dependent on context groups

	**Roman Catholic context**	**Protestant context**	**Non-religiously affilated context**
	**(N = 404**^**a**^**)**	**(N = 130**^**a**^**)**	**(N = 516**^**a**^**)**
Additional physical abuse	125 (45.8%)	47 (47.5%)	151 (44.4%)
Additional psychological abuse	104 (38.1%)	38 (38.4%)	134 (39.4%)
Additional emotional neglect	44 (16.1%)	14 (14.1%)	55 (16.2%)
**Total of victims referring to this topic**	273 (100.0%)	99 (100.0%)	340 (100.0%)

ªTotal of whole context group; see detailed number of victims that referred to single topics in the table.

Table 7 shows the number of cases in which victims reported having been abused by a single offender versus multiple offenders; and, if multiple offenders, whether the assaults were one-time or repeated. Within both the Roman Catholic and the secular groups, the rates reporting a single offender vs. multiple offenders were approximately equal, while in the Protestant group, the rate reporting multiple offenders was lower (35.2%), resulting in a significant difference between the three groups (p = 0.014). Pairwise comparisons showed a significant difference between the Protestant group and the secular group (p = 0.008) and between the Roman Catholic and the secular group (p = 0.048). In all groups, the percentage of victims reporting repeated assaults by multiple offenders was similar, and the percentages reporting repeated assaults were far higher than those reporting just a single act.

**Table 7:** Number of offenders dependent on context group

	**Roman Catholic context**	**Protestant context**	**Non-religiously affiliated context**	**p-value**^**b**^
	**(N = 404**^**a**^**)**	**(N = 130**^**a**^**)**	**(N = 516**^**a**^**)**	
**Number of offenders**				.014
One offender	185 (53.2%)	68 (64.8%)	187 (50.1%)	
Multiple offenders	163 (46.8%)	37 (35.2%)	186 (49.9%)	
**Multiple offenders with one act**	30 (23.8%)	11 (26.2%)	35 (15.8%)	.204
**Multiple offenders with various acts**	96 (76.2%)	31 (73.8%)	137 (84.2%)	.330

ªTotal of whole context group; see detailed number of victims that referred to single topics in the table.

^b^Kruskall-Wallis-Test with statistically significant level p < .05.

Table 8 presents the reported rates of current mental health disorders and problems in psychosocial functioning. Data were collected using categories modeled on the International Classification of Mental Disorders (ICD-10); respondents could indicate problems in more than one category. The majority of victims in all groups (73.6% to 80.2%) reported at least one psychiatric problem. There were no significant group differences in the rates of those who reported any diagnosis or in the rates of those who reported one, two, or three diagnoses. The most common diagnoses reported were depressive episodes, post-traumatic stress syndrome, and anxiety or obsessive-compulsive disorder. It must be noted that as the diagnoses were self-reported and it is not known how they were derived, their validity cannot be verified. A group difference was seen with respect to current psychosocial problems (p = 0.018), with victims in the Protestant group reporting a higher rate of problems overall than victims in either the Roman Catholic group (p = 0.034) or the secular group (p = 0.005). Within each group, the two most commonly reported psychosocial problems were health issues and relationship difficulties.

**Table 8:** Current diagnoses and mental health disorders specified by victims

	**Roman Catholic context**	**Protestant context**	**Non-religiously affilated context**	**p-value**^**b**^
	**(N = 404**^**a**^**)**	**(N = 130**^**a**^**)**	**(N = 516**^**a**^**)**	
**Specified diagnoses**				
Yes	324 (80.2%)	102 (78.5%)	380 (73.6%)	.058
No	80 (19.8%)	28 (21.5%)	136 (26.4%)	
**Number of diagnoses**				
One	64 (15.8%)	20 (15.4%)	88 (17.1%)	.182
Two	21 (5.2%)	7 (5.4%)	35 (6.8%)	
Three	5 (1.2%)	3 (2.3%)	12 (2.3%)	
**Most frequently specified diagnoses**				
First	Depression	Depression	Depression	
	49 (45.0%)	21 (48.8%)	69 (34.3%)	.058
Second	PTSD	Anxiety/obsessive-compulsive disorder	PTSD	
	19 (17.4%)	8 (18.6%)	45 (22.4%)	
Third	Anxiety/ disorder	PTSD	Anxiety/ disorder	
	16 (14.7%)	7 (16.3%)	39 (19.4%)	
**Most frequently specified psychosocial problems**				
First	Health issues	Health issues	Health issues	
	92 (21.6%)	40 (22.6%)	104 (20.8%)	
Second	Relationship and partnership issues	Relationship and partnership issues	Relationship and partnership issues	
	80 (18.8%)	35 (19.8%)	97 (19.4%)	
Third	Flashbacks/intrusion/nightmare	Performance issues	Flashbacks/intrusion/nightmare	
	69 (16.2%)	31 (17.5%)	87 (17.4%)	

ªTotal of whole context group; see detailed number of victims that referred to single topics in the table.

^b^Kruskall-Wallis-Test with statistically significant level p < .05.

### Qualitative results

In addition to the categorical measures described above, data were collected on some aspects of abuse that were analysed qualitatively. Many victims reported that they had been subjected not only to sexual but to physical and emotional abuse. In earlier decades, children’s rights were not very strong, and corporal punishment was a regular practice. For many, the abuse had continued over a long period of time. Offenders were very often in positions of high prestige and power, and victims felt dependent and helpless. Most victims reported that they had received no help, and if they tried to tell someone about the abuse (e.g. family, other adults), they were not taken seriously or were even punished for making the accusation. Disclosure of abuse mostly occurred years after the abuse has been experienced. None had been aware that there were regulatory authorities overseeing the institutions that they should have been able to appeal to.

Some strategies used by offenders were common to all three groups:

► Gaining the victim’s trust by building a close relationship, then leading up to the abuse step by step

► Creating situations where they were alone with the victim, such as private lessons or confession

► Disguising sexual abuse as something educational; e.g., claiming that they were teaching the child about sexuality

Other strategies were specific to religiously affiliated institutions:

► Victims were sometimes encouraged (by their parents or others) to go speak to their priest or pastor about any personal problems they were having, and were abused when they did so

► Religious concepts were sometimes used coercively, such as in threatening children with religious punishment (e.g., “hell) if they did not submit to the abuse; or the sexual abuse was practised as a form of religious ritual

The cases that we looked at had taken place during different decades; and society’s sexual mores, knowledge about sexuality, and handling of abuse have all changed over the years. Victims who had been abused in the 1950s and 1960s were more likely to describe feelings of shame and ignorance about sexuality, whereas those who had been abused in the 1970s were sometimes the target of “reform-minded” pedagogues who viewed sexual abuse not as a problematic or traumatic experience for children but as being helpful for their sexual development.

## Discussion

While much knowledge has come out of the official investigations conducted in different countries in the last decade, sexual abuse in institutions is still an understudied domain. Many studies on child sexual abuse have failed to include an appropriate number of victims of institutional abuse. For example, in a 2010 survey examining prevalence data on sexual abuse in Germany that contacted nearly 12,000 individuals, just 4 respondents – less than 0.001 percent of the total – were former residents of children’s homes [2]. The current study, based on data collected in 2010–2011 through the reporting system established by the Independent Commissioner, represents the largest sample of victims of child sexual abuse in institutions ever studied in Germany. It is also the first to specifically compare sexual abuse within the Roman Catholic, Protestant, and non–religiously affiliated settings, as most earlier reports focused on Roman Catholic institutions only e.g. [41,42].

Sexual abuse was reported for each of these three settings. Based on the testimonials of victims of child sexual abuse in our sample it appears that factors common to all institutions such as group cohesion, hierarchical power structures and dependence [17,42], and credibility bias in favor of authority figures are conducive to the occurrence of repeated sexual abuse over long periods of time, regardless of religious affiliation. Many of the patterns of abuse in all three settings regardless of religious affiliation were similar: victims from all three settings described repeated assaults that were committed by males; one-third overall reported acts of penetration; and over 90% indicated that the abuse had been ongoing. Many victims stated that when they disclosed the abuse to authorities, they were ignored or even punished. Assaults by multiple offenders were reported more often for secular than for religiously affiliated institutions. In all three settings, offenders used the strategies of gaining the victims’ trust, creating situations where they were alone with the victim, and disguising sexual abuse as something educational; all of which have been reported by victims in other studies e.g. [43,44]. Offenders in the religiously affiliated settings additionally exploited situations of trust (e.g., confession), and the use of religious threats to frighten children into submission.

Although females are usually at greater risk for sexual abuse [3], more victims in our sample were male. This can be explained by the fact that generally more boys than girls were sent to boarding schools or residential institutions. This is still true today; as of 2010, two-thirds of the children in youth welfare institutions were boys.

Several studies e.g. [34,45] have revealed a high life prevalence in survivors of institutional child abuse of psychiatric disorders such as depression, anxiety disorders, substance abuse, and PTSD. (In our sample, some victims reported still experiencing PTSD symptoms decades after the abuse had ended.) As not all victims seek treatment and receive diagnoses, the rate in our sample may in fact have underestimated the percentage of victims with such problems. Apart from psychiatric disorders, abuse by a trusted person can also result in psychosocial impairment, affecting a child’s ability to establish relationships and attachments later in life. While some victims show resilience to trauma or are able to overcome its effects [31], others are caught in a complex vicious cycle in which a distorted view of attachment is retained throughout life and has a strong influence on adult relationships [46,47]. In our sample, more females than males described abuse that continued into adulthood. With respect to differences in outcome for victims of religious vs. secular institutions, we found similar rates of psychiatric disorders across all three settings but a higher rate of psychosocial impairment among victims who had been abused in Protestant-run institutions. In line with Max Weber’s sociological theories [48], it is possible that the so-called “Protestant work ethic” played a role in that individuals with higher expectations of success in life might be more distressed by low achievement and hence more likely to report it as an impairment; however, this is only speculation.

The analysis of the qualitative data provided by respondents sheds light on the hardships sometimes faced by children in previous generations. Until relatively recently the problem of child abuse was not discussed openly, and the general public knew little about its prevalence, process, and consequences. Children did not have any special rights; corporal punishment was acceptable (in Germany, it was only legally abolished in 2001); and sex education before the 1970s was rare. Older victims in our sample described dependency, helplessness, and subjection to cruel punishment; and those in institutions with religious affiliations described the additional element of sexual abuse sometimes being disguised as religious ritual, or of the offender threatening the child with religious consequences such as hellfire if he or she resisted.

Most studies of child abuse in institutions or foster care settings have focused on events that took place before the 1980s. The data from the few studies that have looked at more recent situations indicate that the rate of sexual abuse in such settings has decreased in the last decades [12,19], but that children in these environments are still at higher risk of being sexually abused or otherwise maltreated than the population at large [49-52]. There may also still be a lack of awareness and understanding of the problem by those in authority. The government-sponsored reappraisal program and the findings described in this report serve several important functions. First, the program gave victims of previous generations, many of whom had never before revealed the abuse to anyone, the opportunity to speak out about what had happened to them. Second, the political process that was initiated through the program has been designed to improve situation of victims of child sexual abuse (e.g. by assisting victims to get access to effective therapies). Third, the findings showed that past child sexual abuse in institutions was associated not with any particular religious affiliation but with institutions in general. Finally, while the rights of children and the quality of education provided in institutions have improved over the last few decades, advocates for children’s welfare can still learn from these experiences and can use them to strengthen the implementation of prevention and intervention strategies. Although the publicity around the program has led to greater public awareness of child sexual abuse in institutions, the problem may still sometimes be underestimated. In a German study [17] that surveyed 1,128 principals, 702 counsellors, and 736 directors of institutions, merely 3.1% of respondents could cite even a suspected case of sexual abuse within the last three years, which is surprising. However, it is not clear whether this finding reflects true prevalence rates or is skewed by study limitations (e.g., there was no validation of information, and the individuals who agreed to participate were self-selected).

Several important limitations of this study must be acknowledged. First, as the individuals who provided data were self-selected, the sample may not have been representative of the overall population. Victims who made use of the reporting system may have been particularly motivated to do so, and it is possible that our data represent a sub-sample of severely traumatized individuals. Thus, we cannot definitively conclude from our data that the patterns of abuse we were told about did in fact commonly occur. However, the size of the sample and the fact that our results are consistent with those obtained by different international commissions [7,12] support the likelihood that they paint a realistic picture of the conditions in German institutions for the periods that most victims described. Second, apart from demographic data, information was not collected in any standardized way; rather, victims could simply share whatever was relevant to them. It is important to keep this aspect in mind when analyzing and interpreting the findings, because different amounts of information were obtained for different categories of data. Third, there was no independent validation of the information provided. However, as the liaison office had no authority to provide any form of compensation, callers had no financial incentive to invent or exaggerate any of the information that they provided. These limitations were imposed by the confidential nature of the issue under investigation, by the ethical requirement of maintaining victims’ privacy, and by the duty of the Independent Commissioner. Despite these limitations, the method of data collection used in this study has provided valuable exploratory data that can serve as a starting point for building hypotheses. Any hypothesis would need to be tested via representative sampling using standardized interviews or questionnaires, but it must be recognized that those approaches involve asking leading questions that may pressure some participants into making statements that do not fully represent their personal experience.

With respect to institutional affiliations, our results show that sexual abuse is not a problem specific to Roman Catholic settings or to religiously affiliated settings in general, but rather that the risk to children is increased in any institution, regardless of affiliation. Given the prevalence and serious consequence of child sexual abuse in all types of institutions, there is a critical need for effective prevention and intervention strategies, although there have been changes within the last decades concerning rights of children, accepted disciplinary methods, awareness of child sexual abuse. First, professionals who work with children and adolescents should be educated through workshops or e-learning platforms about risks, warning signals, and intervention strategies, as well the consequences of child sexual abuse. E-learning platforms are an effective way to educate a large group of people at the same time. The groups to be targeted include psychotherapists, social workers, teachers, priests/pastors, and educators, as well as physicians since sexual abuse victims may experience problems affecting their physical health [49]. Second, prevention strategies such as guiding principles, supervision, the handling of complaints, and standard processes when suspicion is raised should be implemented in institutions. Third, professionals such as psychiatrists and psychotherapists should be better trained in trauma therapy, and should be made more aware of the occurrence of PTSD symptoms in older patients. In a German survey of more than 2000 psychotherapists [17], respondents said that on average, 22% of their patients had experienced sexual abuse, 38% had started therapy with the aim of overcoming this experience, and 43% had disclosed abuse during the course of the therapy; yet more than half of these therapists did not feel qualified to treat patients with a background of sexual abuse.

## Conclusion

This study is the first to examine patterns and consequences of child sexual abuse in different types of institutions based on a large sample. Based on the information provided by respondents, the nature of institutional structures and overall perceptions of the rights of children rather appeared to be a factor in the prevalence and nature of child sexual abuse than the religious affiliation of institutions. Severe sexual abuse in institutions appears to have decreased over the past decades, but there is still the need for better understanding of it and for the implementation of prevention and intervention strategies. The exploratory data arising from this study may serve as a starting point for building hypotheses.

## Competing interests

NS, JMF, AS, LK, TS, and MR conducted research for the Independent Commissioner on Cases of Child Abuse in Germany. In addition, JMF is a member of the advisory board of the Independent Commissioner. JMF and HL received a grant from the Ministry of Research and Education to develop an e-learning platform for medical-therapeutic and educational professions to prevent sexual abuse of children. The Pontifical Gregorian University (Rome) and the Catholic Diocese of Munich and Freising (Germany) jointly commissioned JMF and HL to develop an e-learning platform for pastoral professions for the prevention of sexual child abuse within the Roman Catholic Church.

## Authors’ contributions

JMF developed the idea of the study and was responsible for obtaining its funding. JMF, NS, and LK were responsible for the design of the study, the web-based documentary pattern, and the data collection; AS developed the web-based platform; JMF, NS, MR, TS, and LK were responsible for the statistical analysis of data and interpretation of the results; and HL was involved in revising the manuscript critically. All authors read and approved the final manuscript.

## Pre-publication history

The pre-publication history for this paper can be accessed here:

http://www.biomedcentral.com/1471-2458/14/282/prepub
